# Supplementary material for: Modeling glioblastoma heterogeneity as a dynamic network of cell states
Source: Mol Syst Biol. 2021 Sep 16;17(9):e10105. doi: 10.15252/msb.202010105 (PMC8444284; doi:10.15252/msb.202010105)
Supplement: Supplementary file 6 — Source Data for Figure 5 [file MSB-17-e10105-s004.zip › Figure5A_sourcedata/GSEA_3071/nosigdb_stateI.GseaPreranked.1622795145664/GAROFANO_IAVARONE_PPR_SUBTYPE.html]

Details for gene set GAROFANO\_IAVARONE\_PPR\_SUBTYPE[GSEA]

|  || Dataset | state13071 |
| Phenotype | NoPhenotypeAvailable |
| Upregulated in class | na\_neg |
| GeneSet | GAROFANO\_IAVARONE\_PPR\_SUBTYPE |
| Enrichment Score (ES) | -0.54164255 |
| Normalized Enrichment Score (NES) | -3.267831 |
| Nominal p-value | 0.0 |
| FDR q-value | 0.0 |
| FWER p-Value | 0.0 |
Table: GSEA Results Summary

  

Fig 1: Enrichment plot: GAROFANO\_IAVARONE\_PPR\_SUBTYPE      
 Profile of the Running ES Score & Positions of GeneSet Members on the Rank Ordered List

  

| PROBE | GENE SYMBOL | GENE\_TITLE | RANK IN GENE LIST | RANK METRIC SCORE | RUNNING ES | CORE ENRICHMENT || 1 | EPB41 |  |  | 536 | 0.488 | -0.1620 | No |
| 2 | FMNL3 |  |  | 685 | 0.419 | -0.2049 | No |
| 3 | ASXL3 |  |  | 910 | 0.348 | -0.2717 | No |
| 4 | BSCL2 |  |  | 1106 | 0.305 | -0.3298 | No |
| 5 | PASK |  |  | 1323 | 0.265 | -0.3946 | No |
| 6 | FCHSD2 |  |  | 1402 | 0.252 | -0.4170 | No |
| 7 | MSH2 |  |  | 1438 | -0.252 | -0.4261 | No |
| 8 | CYTH2 |  |  | 1443 | -0.252 | -0.4257 | No |
| 9 | CEP152 |  |  | 1446 | -0.253 | -0.4246 | No |
| 10 | RSBN1L |  |  | 1447 | -0.253 | -0.4230 | No |
| 11 | CTCF |  |  | 1450 | -0.253 | -0.4219 | No |
| 12 | HNRNPH1 |  |  | 1500 | -0.257 | -0.4353 | No |
| 13 | CREBBP |  |  | 1518 | -0.259 | -0.4388 | No |
| 14 | NR2C2AP |  |  | 1527 | -0.260 | -0.4396 | No |
| 15 | MIER3 |  |  | 1533 | -0.261 | -0.4394 | No |
| 16 | ATG4D |  |  | 1537 | -0.261 | -0.4386 | No |
| 17 | RBBP4 |  |  | 1549 | -0.262 | -0.4403 | No |
| 18 | CHAF1B |  |  | 1554 | -0.262 | -0.4397 | No |
| 19 | ADNP2 |  |  | 1585 | -0.266 | -0.4472 | No |
| 20 | GALE |  |  | 1592 | -0.267 | -0.4473 | No |
| 21 | ERI2 |  |  | 1593 | -0.267 | -0.4455 | No |
| 22 | SAE1 |  |  | 1638 | -0.273 | -0.4573 | No |
| 23 | SMC3 |  |  | 1650 | -0.274 | -0.4589 | No |
| 24 | RAD54L |  |  | 1702 | -0.280 | -0.4728 | No |
| 25 | WIZ |  |  | 1704 | -0.280 | -0.4712 | No |
| 26 | PNRC2 |  |  | 1731 | -0.284 | -0.4774 | No |
| 27 | KIF2C |  |  | 1735 | -0.284 | -0.4764 | No |
| 28 | RP9 |  |  | 1748 | -0.286 | -0.4782 | No |
| 29 | CCNB2 |  |  | 1757 | -0.288 | -0.4788 | No |
| 30 | ZNF273 |  |  | 1760 | -0.288 | -0.4775 | No |
| 31 | CENPJ |  |  | 1768 | -0.290 | -0.4777 | No |
| 32 | EXOSC9 |  |  | 1777 | -0.291 | -0.4782 | No |
| 33 | TRIM28 |  |  | 1789 | -0.292 | -0.4797 | No |
| 34 | ZNF593 |  |  | 1793 | -0.293 | -0.4787 | No |
| 35 | CDK14 |  |  | 1798 | -0.293 | -0.4780 | No |
| 36 | C2CD4C |  |  | 1809 | -0.295 | -0.4791 | No |
| 37 | HNRNPA2B1 |  |  | 1819 | -0.296 | -0.4799 | No |
| 38 | TROAP |  |  | 1821 | -0.296 | -0.4783 | No |
| 39 | RAD51 |  |  | 1845 | -0.299 | -0.4834 | No |
| 40 | DOT1L |  |  | 1850 | -0.299 | -0.4826 | No |
| 41 | CASP8AP2 |  |  | 1857 | -0.300 | -0.4825 | No |
| 42 | PTBP1 |  |  | 1934 | -0.311 | -0.5039 | No |
| 43 | KPNA2 |  |  | 1944 | -0.312 | -0.5046 | No |
| 44 | CDC25A |  |  | 1953 | -0.313 | -0.5050 | No |
| 45 | ZNF92 |  |  | 1962 | -0.313 | -0.5054 | No |
| 46 | ZNF22 |  |  | 1967 | -0.314 | -0.5045 | No |
| 47 | VEZF1 |  |  | 1970 | -0.314 | -0.5031 | No |
| 48 | DGCR8 |  |  | 1991 | -0.317 | -0.5071 | No |
| 49 | CEP78 |  |  | 2000 | -0.317 | -0.5075 | No |
| 50 | INCENP |  |  | 2018 | -0.320 | -0.5106 | No |
| 51 | SUV39H2 |  |  | 2041 | -0.324 | -0.5153 | No |
| 52 | DNA2 |  |  | 2057 | -0.327 | -0.5177 | No |
| 53 | SRRT |  |  | 2077 | -0.331 | -0.5214 | No |
| 54 | ZNF93 |  |  | 2095 | -0.333 | -0.5244 | No |
| 55 | CHEK1 |  |  | 2108 | -0.336 | -0.5259 | No |
| 56 | SP4 |  |  | 2110 | -0.336 | -0.5240 | No |
| 57 | C18orf54 |  |  | 2128 | -0.339 | -0.5270 | No |
| 58 | PLK4 |  |  | 2167 | -0.346 | -0.5364 | No |
| 59 | ZNF85 |  |  | 2185 | -0.349 | -0.5393 | Yes |
| 60 | HNRNPL |  |  | 2190 | -0.350 | -0.5382 | Yes |
| 61 | BRD7 |  |  | 2193 | -0.350 | -0.5365 | Yes |
| 62 | POC1A |  |  | 2199 | -0.351 | -0.5358 | Yes |
| 63 | PLK1 |  |  | 2206 | -0.353 | -0.5353 | Yes |
| 64 | PKMYT1 |  |  | 2214 | -0.356 | -0.5351 | Yes |
| 65 | CENPA |  |  | 2220 | -0.357 | -0.5342 | Yes |
| 66 | ILF3 |  |  | 2222 | -0.358 | -0.5322 | Yes |
| 67 | WDR62 |  |  | 2233 | -0.360 | -0.5329 | Yes |
| 68 | SMC1A |  |  | 2240 | -0.361 | -0.5323 | Yes |
| 69 | POLE |  |  | 2252 | -0.364 | -0.5333 | Yes |
| 70 | POLQ |  |  | 2253 | -0.364 | -0.5309 | Yes |
| 71 | PAFAH1B3 |  |  | 2264 | -0.365 | -0.5316 | Yes |
| 72 | ZNF107 |  |  | 2272 | -0.366 | -0.5313 | Yes |
| 73 | NUCKS1 |  |  | 2278 | -0.368 | -0.5304 | Yes |
| 74 | DEPDC1B |  |  | 2288 | -0.370 | -0.5307 | Yes |
| 75 | FUS |  |  | 2300 | -0.373 | -0.5317 | Yes |
| 76 | CKS1B |  |  | 2332 | -0.380 | -0.5387 | Yes |
| 77 | BUB1B |  |  | 2335 | -0.381 | -0.5368 | Yes |
| 78 | ING1 |  |  | 2337 | -0.382 | -0.5346 | Yes |
| 79 | MCM2 |  |  | 2349 | -0.383 | -0.5354 | Yes |
| 80 | CKAP2 |  |  | 2357 | -0.385 | -0.5350 | Yes |
| 81 | ZWILCH |  |  | 2358 | -0.385 | -0.5325 | Yes |
| 82 | MSH6 |  |  | 2362 | -0.386 | -0.5309 | Yes |
| 83 | CCNF |  |  | 2370 | -0.387 | -0.5304 | Yes |
| 84 | STIL |  |  | 2374 | -0.388 | -0.5288 | Yes |
| 85 | POLA1 |  |  | 2376 | -0.388 | -0.5265 | Yes |
| 86 | RBMX |  |  | 2378 | -0.388 | -0.5243 | Yes |
| 87 | MRTO4 |  |  | 2393 | -0.391 | -0.5260 | Yes |
| 88 | EXOSC2 |  |  | 2400 | -0.392 | -0.5252 | Yes |
| 89 | CENPW |  |  | 2402 | -0.393 | -0.5230 | Yes |
| 90 | NEIL3 |  |  | 2413 | -0.395 | -0.5234 | Yes |
| 91 | BUB1 |  |  | 2416 | -0.396 | -0.5214 | Yes |
| 92 | HAUS1 |  |  | 2423 | -0.397 | -0.5206 | Yes |
| 93 | H2AFV |  |  | 2431 | -0.398 | -0.5202 | Yes |
| 94 | HNRNPU |  |  | 2434 | -0.398 | -0.5181 | Yes |
| 95 | XPA |  |  | 2440 | -0.400 | -0.5170 | Yes |
| 96 | ZNF738 |  |  | 2441 | -0.400 | -0.5144 | Yes |
| 97 | POLA2 |  |  | 2452 | -0.401 | -0.5148 | Yes |
| 98 | CENPO |  |  | 2460 | -0.404 | -0.5143 | Yes |
| 99 | BLM |  |  | 2469 | -0.404 | -0.5141 | Yes |
| 100 | KIF11 |  |  | 2488 | -0.410 | -0.5169 | Yes |
| 101 | HAUS8 |  |  | 2494 | -0.412 | -0.5157 | Yes |
| 102 | ZNF519 |  |  | 2504 | -0.416 | -0.5157 | Yes |
| 103 | SNRPA |  |  | 2515 | -0.417 | -0.5161 | Yes |
| 104 | SPAG5 |  |  | 2519 | -0.418 | -0.5142 | Yes |
| 105 | RACGAP1 |  |  | 2522 | -0.419 | -0.5121 | Yes |
| 106 | BAZ1B |  |  | 2538 | -0.425 | -0.5139 | Yes |
| 107 | MEX3A |  |  | 2559 | -0.429 | -0.5172 | Yes |
| 108 | ZNF100 |  |  | 2567 | -0.431 | -0.5165 | Yes |
| 109 | H2AFZ |  |  | 2572 | -0.433 | -0.5149 | Yes |
| 110 | SSRP1 |  |  | 2580 | -0.434 | -0.5141 | Yes |
| 111 | ELAVL1 |  |  | 2588 | -0.436 | -0.5134 | Yes |
| 112 | KNTC1 |  |  | 2613 | -0.443 | -0.5179 | Yes |
| 113 | RMI1 |  |  | 2616 | -0.444 | -0.5156 | Yes |
| 114 | NCAPG2 |  |  | 2621 | -0.445 | -0.5138 | Yes |
| 115 | FANCB |  |  | 2623 | -0.446 | -0.5112 | Yes |
| 116 | HIRIP3 |  |  | 2635 | -0.449 | -0.5116 | Yes |
| 117 | DUT |  |  | 2640 | -0.450 | -0.5099 | Yes |
| 118 | DCTPP1 |  |  | 2644 | -0.451 | -0.5078 | Yes |
| 119 | MASTL |  |  | 2646 | -0.452 | -0.5051 | Yes |
| 120 | EXOSC8 |  |  | 2659 | -0.456 | -0.5058 | Yes |
| 121 | CCNA2 |  |  | 2667 | -0.462 | -0.5049 | Yes |
| 122 | RFWD3 |  |  | 2668 | -0.462 | -0.5018 | Yes |
| 123 | SKA2 |  |  | 2672 | -0.464 | -0.4997 | Yes |
| 124 | FIGNL1 |  |  | 2679 | -0.465 | -0.4985 | Yes |
| 125 | MND1 |  |  | 2682 | -0.468 | -0.4960 | Yes |
| 126 | KIF22 |  |  | 2683 | -0.468 | -0.4929 | Yes |
| 127 | TOP2B |  |  | 2686 | -0.469 | -0.4904 | Yes |
| 128 | CDCA8 |  |  | 2695 | -0.470 | -0.4897 | Yes |
| 129 | MCM6 |  |  | 2699 | -0.471 | -0.4875 | Yes |
| 130 | PRC1 |  |  | 2705 | -0.472 | -0.4860 | Yes |
| 131 | FANCD2 |  |  | 2707 | -0.474 | -0.4831 | Yes |
| 132 | CENPN |  |  | 2708 | -0.474 | -0.4800 | Yes |
| 133 | MELK |  |  | 2709 | -0.474 | -0.4768 | Yes |
| 134 | NDC80 |  |  | 2723 | -0.478 | -0.4777 | Yes |
| 135 | EXO1 |  |  | 2728 | -0.479 | -0.4757 | Yes |
| 136 | BRIP1 |  |  | 2739 | -0.482 | -0.4756 | Yes |
| 137 | TIMELESS |  |  | 2752 | -0.487 | -0.4761 | Yes |
| 138 | CENPF |  |  | 2761 | -0.490 | -0.4753 | Yes |
| 139 | TUBA1A |  |  | 2771 | -0.495 | -0.4748 | Yes |
| 140 | BRCA2 |  |  | 2772 | -0.495 | -0.4715 | Yes |
| 141 | NUF2 |  |  | 2781 | -0.497 | -0.4707 | Yes |
| 142 | TUBA1B |  |  | 2785 | -0.498 | -0.4683 | Yes |
| 143 | SKA1 |  |  | 2787 | -0.498 | -0.4653 | Yes |
| 144 | DTYMK |  |  | 2798 | -0.501 | -0.4651 | Yes |
| 145 | WDR76 |  |  | 2800 | -0.501 | -0.4621 | Yes |
| 146 | HMGB1 |  |  | 2806 | -0.503 | -0.4603 | Yes |
| 147 | RBL1 |  |  | 2808 | -0.503 | -0.4573 | Yes |
| 148 | HNRNPAB |  |  | 2816 | -0.508 | -0.4561 | Yes |
| 149 | LSM5 |  |  | 2820 | -0.509 | -0.4536 | Yes |
| 150 | ASF1B |  |  | 2824 | -0.510 | -0.4512 | Yes |
| 151 | DSCC1 |  |  | 2828 | -0.512 | -0.4487 | Yes |
| 152 | TMEM97 |  |  | 2834 | -0.514 | -0.4468 | Yes |
| 153 | SPC24 |  |  | 2836 | -0.514 | -0.4437 | Yes |
| 154 | HMGB3 |  |  | 2839 | -0.516 | -0.4409 | Yes |
| 155 | TOP2A |  |  | 2842 | -0.517 | -0.4381 | Yes |
| 156 | ZNF43 |  |  | 2844 | -0.517 | -0.4350 | Yes |
| 157 | E2F8 |  |  | 2847 | -0.519 | -0.4322 | Yes |
| 158 | LIG1 |  |  | 2848 | -0.520 | -0.4288 | Yes |
| 159 | CKAP2L |  |  | 2857 | -0.523 | -0.4278 | Yes |
| 160 | ECT2 |  |  | 2858 | -0.523 | -0.4243 | Yes |
| 161 | NUDT1 |  |  | 2859 | -0.524 | -0.4208 | Yes |
| 162 | CDK2 |  |  | 2863 | -0.526 | -0.4183 | Yes |
| 163 | TRIP13 |  |  | 2865 | -0.526 | -0.4151 | Yes |
| 164 | HJURP |  |  | 2869 | -0.527 | -0.4125 | Yes |
| 165 | ANP32E |  |  | 2877 | -0.530 | -0.4112 | Yes |
| 166 | ARHGAP11A |  |  | 2921 | -0.556 | -0.4207 | Yes |
| 167 | ATAD5 |  |  | 2935 | -0.563 | -0.4210 | Yes |
| 168 | FANCA |  |  | 2937 | -0.565 | -0.4176 | Yes |
| 169 | CENPH |  |  | 2945 | -0.569 | -0.4160 | Yes |
| 170 | DSN1 |  |  | 2949 | -0.571 | -0.4131 | Yes |
| 171 | MAD2L2 |  |  | 2952 | -0.573 | -0.4099 | Yes |
| 172 | KIF4A |  |  | 2953 | -0.574 | -0.4061 | Yes |
| 173 | TACC3 |  |  | 2956 | -0.575 | -0.4029 | Yes |
| 174 | KIF23 |  |  | 2961 | -0.579 | -0.4003 | Yes |
| 175 | NCAPH |  |  | 2974 | -0.585 | -0.4001 | Yes |
| 176 | POP7 |  |  | 2980 | -0.588 | -0.3978 | Yes |
| 177 | SMC2 |  |  | 2984 | -0.592 | -0.3948 | Yes |
| 178 | SKA3 |  |  | 2985 | -0.592 | -0.3909 | Yes |
| 179 | TPX2 |  |  | 3006 | -0.606 | -0.3930 | Yes |
| 180 | RTKN2 |  |  | 3020 | -0.613 | -0.3930 | Yes |
| 181 | DNMT1 |  |  | 3035 | -0.622 | -0.3932 | Yes |
| 182 | PARP1 |  |  | 3039 | -0.626 | -0.3899 | Yes |
| 183 | ADD2 |  |  | 3054 | -0.638 | -0.3900 | Yes |
| 184 | AURKB |  |  | 3056 | -0.639 | -0.3861 | Yes |
| 185 | LMNB2 |  |  | 3061 | -0.644 | -0.3831 | Yes |
| 186 | NCAPD3 |  |  | 3064 | -0.646 | -0.3794 | Yes |
| 187 | CDKN1B |  |  | 3065 | -0.646 | -0.3751 | Yes |
| 188 | DPF1 |  |  | 3071 | -0.651 | -0.3724 | Yes |
| 189 | FANCI |  |  | 3074 | -0.656 | -0.3686 | Yes |
| 190 | CDC45 |  |  | 3081 | -0.661 | -0.3661 | Yes |
| 191 | GTSE1 |  |  | 3083 | -0.663 | -0.3620 | Yes |
| 192 | ZNF704 |  |  | 3086 | -0.665 | -0.3582 | Yes |
| 193 | TMPO |  |  | 3088 | -0.666 | -0.3541 | Yes |
| 194 | SAP30 |  |  | 3096 | -0.671 | -0.3518 | Yes |
| 195 | MAD2L1 |  |  | 3099 | -0.675 | -0.3480 | Yes |
| 196 | NASP |  |  | 3106 | -0.682 | -0.3453 | Yes |
| 197 | RFC3 |  |  | 3108 | -0.683 | -0.3411 | Yes |
| 198 | KIFC1 |  |  | 3110 | -0.684 | -0.3369 | Yes |
| 199 | LSM4 |  |  | 3115 | -0.688 | -0.3336 | Yes |
| 200 | DBF4 |  |  | 3117 | -0.690 | -0.3293 | Yes |
| 201 | LMNB1 |  |  | 3121 | -0.692 | -0.3256 | Yes |
| 202 | POLD3 |  |  | 3122 | -0.695 | -0.3210 | Yes |
| 203 | PGAP1 |  |  | 3123 | -0.696 | -0.3164 | Yes |
| 204 | SMC4 |  |  | 3126 | -0.699 | -0.3124 | Yes |
| 205 | CENPK |  |  | 3127 | -0.700 | -0.3078 | Yes |
| 206 | MCM5 |  |  | 3132 | -0.706 | -0.3043 | Yes |
| 207 | GINS1 |  |  | 3137 | -0.713 | -0.3008 | Yes |
| 208 | CCDC88A |  |  | 3138 | -0.714 | -0.2961 | Yes |
| 209 | GMNN |  |  | 3140 | -0.719 | -0.2917 | Yes |
| 210 | KIF15 |  |  | 3142 | -0.723 | -0.2872 | Yes |
| 211 | GAB1 |  |  | 3144 | -0.724 | -0.2827 | Yes |
| 212 | CDK1 |  |  | 3149 | -0.727 | -0.2791 | Yes |
| 213 | HNRNPD |  |  | 3157 | -0.738 | -0.2764 | Yes |
| 214 | MKI67 |  |  | 3158 | -0.738 | -0.2715 | Yes |
| 215 | PRIM1 |  |  | 3160 | -0.739 | -0.2669 | Yes |
| 216 | TK1 |  |  | 3162 | -0.745 | -0.2623 | Yes |
| 217 | DNAJC9 |  |  | 3167 | -0.747 | -0.2585 | Yes |
| 218 | STMN1 |  |  | 3171 | -0.751 | -0.2545 | Yes |
| 219 | CDT1 |  |  | 3179 | -0.758 | -0.2516 | Yes |
| 220 | BRCA1 |  |  | 3185 | -0.765 | -0.2481 | Yes |
| 221 | CDCA2 |  |  | 3189 | -0.767 | -0.2440 | Yes |
| 222 | XRCC2 |  |  | 3197 | -0.777 | -0.2410 | Yes |
| 223 | PXMP2 |  |  | 3200 | -0.782 | -0.2364 | Yes |
| 224 | BARD1 |  |  | 3201 | -0.783 | -0.2312 | Yes |
| 225 | CDCA7 |  |  | 3203 | -0.785 | -0.2263 | Yes |
| 226 | CDC6 |  |  | 3204 | -0.785 | -0.2211 | Yes |
| 227 | BIRC5 |  |  | 3208 | -0.793 | -0.2168 | Yes |
| 228 | RRM1 |  |  | 3215 | -0.801 | -0.2133 | Yes |
| 229 | SPC25 |  |  | 3219 | -0.806 | -0.2089 | Yes |
| 230 | UBE2C |  |  | 3225 | -0.812 | -0.2051 | Yes |
| 231 | NUSAP1 |  |  | 3237 | -0.825 | -0.2030 | Yes |
| 232 | FOXM1 |  |  | 3238 | -0.826 | -0.1975 | Yes |
| 233 | HMGN2 |  |  | 3242 | -0.833 | -0.1929 | Yes |
| 234 | RFC2 |  |  | 3245 | -0.839 | -0.1880 | Yes |
| 235 | SALL3 |  |  | 3248 | -0.844 | -0.1830 | Yes |
| 236 | DTL |  |  | 3249 | -0.848 | -0.1774 | Yes |
| 237 | LCORL |  |  | 3256 | -0.859 | -0.1736 | Yes |
| 238 | CENPM |  |  | 3269 | -0.881 | -0.1714 | Yes |
| 239 | CHAF1A |  |  | 3270 | -0.882 | -0.1656 | Yes |
| 240 | CDCA5 |  |  | 3273 | -0.885 | -0.1603 | Yes |
| 241 | FEN1 |  |  | 3278 | -0.890 | -0.1557 | Yes |
| 242 | H2AFX |  |  | 3282 | -0.898 | -0.1507 | Yes |
| 243 | HELLS |  |  | 3284 | -0.901 | -0.1450 | Yes |
| 244 | KLHL23 |  |  | 3287 | -0.907 | -0.1396 | Yes |
| 245 | RANBP1 |  |  | 3290 | -0.911 | -0.1342 | Yes |
| 246 | ZWINT |  |  | 3296 | -0.925 | -0.1296 | Yes |
| 247 | PCNA |  |  | 3319 | -0.963 | -0.1300 | Yes |
| 248 | UBE2T |  |  | 3330 | -0.979 | -0.1266 | Yes |
| 249 | MYBL2 |  |  | 3340 | -1.006 | -0.1227 | Yes |
| 250 | MCM4 |  |  | 3341 | -1.006 | -0.1160 | Yes |
| 251 | EZH2 |  |  | 3342 | -1.015 | -0.1093 | Yes |
| 252 | RAD51AP1 |  |  | 3345 | -1.030 | -0.1031 | Yes |
| 253 | MCM10 |  |  | 3356 | -1.050 | -0.0992 | Yes |
| 254 | HIST2H2AC |  |  | 3363 | -1.077 | -0.0940 | Yes |
| 255 | ESCO2 |  |  | 3367 | -1.088 | -0.0877 | Yes |
| 256 | NCAPG |  |  | 3370 | -1.098 | -0.0810 | Yes |
| 257 | MCM7 |  |  | 3384 | -1.147 | -0.0774 | Yes |
| 258 | GINS2 |  |  | 3386 | -1.156 | -0.0701 | Yes |
| 259 | HMGB2 |  |  | 3390 | -1.184 | -0.0632 | Yes |
| 260 | CLSPN |  |  | 3391 | -1.184 | -0.0553 | Yes |
| 261 | UBE2S |  |  | 3396 | -1.205 | -0.0486 | Yes |
| 262 | FAM111B |  |  | 3402 | -1.238 | -0.0419 | Yes |
| 263 | ATAD2 |  |  | 3415 | -1.278 | -0.0371 | Yes |
| 264 | RRM2 |  |  | 3420 | -1.297 | -0.0298 | Yes |
| 265 | MAP2 |  |  | 3422 | -1.305 | -0.0214 | Yes |
| 266 | TYMS |  |  | 3436 | -1.391 | -0.0162 | Yes |
| 267 | HIST1H1B |  |  | 3467 | -1.770 | -0.0138 | Yes |
| 268 | SOX11 |  |  | 3469 | -1.784 | -0.0022 | Yes |
| 269 | HIST1H4C |  |  | 3493 | -2.248 | 0.0056 | Yes |
Table: GSEA details [plain text format]

  

Fig 2: GAROFANO\_IAVARONE\_PPR\_SUBTYPE: Random ES distribution      
 Gene set null distribution of ES for **GAROFANO\_IAVARONE\_PPR\_SUBTYPE**
